# Enhancing Home-Based Exercise Therapy with Telerehabilitation in Mild Adolescent Idiopathic Scoliosis: A Randomized Controlled Trial

**DOI:** 10.3390/healthcare14010019

**Published:** 2025-12-21

**Authors:** Zuhal Didem Takinacı, Meltem Çelik, Şeyda Yıldız, Mehmet Ali Talmaç, Raziye Dut

**Affiliations:** 1 Physiotherapy and Rehabilitation Department, University of Health Sciences, Istanbul 34668, Turkey; mcelik@ortopediatri.com.tr (M.Ç.); seyda.yildiz@istun.edu.tr (Ş.Y.); 2 Physiotherapy and Rehabilitation Department, Ortopediatri Academy of Pediatric Orthopedics, Istanbul 343449, Turkey; 3 Physiotherapy and Rehabilitation Department, Istanbul Health and Technology University, Istanbul 34275, Turkey; 4 Orthopedics and Traumatology Department, University of Health Sciences, Istanbul 34668, Turkey; 251003004@ogrenci.sbu.edu.tr; 5 Department of Pediatrics and Adolescent Health Medicine, Istanbul Training and Research Hospital, Istanbul 34098, Turkey; raziyemektup@yahoo.com

**Keywords:** adolescent idiopathic scoliosis, telerehabilitation, home-based exercise

## Abstract

**Background and Objectives:** Adolescent idiopathic scoliosis (AIS) is a three-dimensional spinal deformity that affects postural alignment, function, and quality of life. Telerehabilitation has emerged as a promising approach to enhance accessibility and continuity of exercise-based treatment in AIS. This study aimed to compare the effects of telerehabilitation-supported home exercise programs with standard home exercises on posture, pain, body image, and quality of life in adolescents with mild AIS. **Materials and Methods:** Forty adolescents aged 10–18 years with mild AIS (Cobb angle 10–25°, Risser 0–3) were randomly assigned to two groups: study (n = 20) and control (n = 20). Both groups performed an 8-week home-based exercise program. The study group additionally received weekly online supervision by a physiotherapist. Outcomes included pain severity (VAS), posture (New York Posture Assessment Scale), body image (Walter Reed Visual Assessment Scale), and quality of life (SRS-22 questionnaire). Statistical analyses were performed using non-parametric tests, with a significance level of *p* < 0.05. **Results:** Twenty-nine participants completed the study (15 in the study group, 14 in the control group). Significant improvements were observed in the study group in SRS-22 total, pain, and function subscores, as well as posture scores (*p* < 0.05). In the control group, only the satisfaction subscore improved significantly (*p* < 0.05). No significant changes were detected in body image (WRVAS) in either group. Between-group comparisons showed greater overall clinical gains in the study group despite similar exercise adherence rates. **Conclusions:** Supervised telerehabilitation enhances the effectiveness of home-based exercise programs in adolescents with mild AIS by improving postural alignment, reducing pain, and increasing functional capacity and quality of life. Telerehabilitation represents an accessible and efficient complementary strategy for managing AIS when in-person supervision is limited.

## 1. Introduction

Adolescent idiopathic scoliosis (AIS) is a three-dimensional deformity characterized by a curvature of the spine greater than 10 degrees in the coronal plane, a deterioration in the physiological curvature in the sagittal plane and accompanying rotation in the axial plane [1]. AIS is mostly seen at the thoracic level, less frequently at the lumbar and thoracolumbar levels, and is the most common form of structural scoliosis, accounting for approximately 80% of all scoliosis cases [2].

In AIS, the problem with spinal alignment affects the bones and soft tissue in the entire body; the resulting deformities, such as postural disorders and loss of flexibility of the spine, cause problems such as cosmetic concerns, functional losses, and pain in adolescents. In severe cases, respiratory functions and lung capacity decrease, and the patient’s quality of life is affected by this condition [3]. Different treatment approaches are recommended for the prevention and management of all these problems. When determining the appropriate treatment option, the type, shape, degree of the curve, and the patient’s characteristics should be taken into consideration. To stop or prevent the progression of the curve or to combat the complications experienced, different exercise approaches for scoliosis, the use of corsets, and, in more severe cases, surgical methods are the basic/main treatment options [4]. For moderate curves below 40°, special exercises and corsets are expressed as first-line treatments to control scoliosis without the need for surgery; therefore, various exercise approaches are frequently used at all stages of AIS treatment [2,3,5].

When the literature is examined, it has been reported that exercises for AIS are often recommended to prevent the progression of the curve, correct postural behavior and provide awareness, increase neuromotor control of the spine, increase spinal and thoracic flexibility, increase muscle strength by optimizing muscle balance, and achieve body symmetry [4,6]. In the treatment of scoliosis, specific exercises are usually given to the individual according to the shape, type, and degree of the curve. Therapeutic exercises include breathing exercises, self-correction exercises (mental imagery, exteroceptive, proprioceptive stimuli, and mirror control), and stabilization exercises (physical exercise, yoga, pilates, and core stabilization) [7,8]. Additionally recent home-based exercise studies in adolescents with musculoskeletal and spinal conditions have also highlighted the importance of adherence and remote monitoring to maintain exercise intensity and frequency [9,10,11].

Telerehabilitation-based interventions are preferred to continue treatment when patients’ access to rehabilitation services is restricted for various reasons. In adolescents with musculoskeletal and spinal disorders, recent telerehabilitation and telerehabilitation-supported home-based exercise programs have been reported to be feasible and to yield outcomes comparable to, and in some cases better than, traditional face-to-face rehabilitation, while supporting exercise adherence and self-management [12,13,14].

There are limited studies comparing telerehabilitation with home exercises; however, recent studies have shown that telerehabilitation may provide more positive results in terms of respiratory function, flexibility and participation [15,16]. These findings reveal that telerehabilitation may positively affect exercise compliance and clinical outcomes in AIS; however, due to the small number of studies and the variety of exercises applied in the studies, the number of comparative data regarding the application of exercises for scoliosis via telerehabilitation remains quite limited. In addition, the difference between the results obtained when the home programs are completely applied by oneself and the results obtained when a physiotherapist guides them remotely via telerehabilitation has not been fully established. Additionally, to our knowledge, there are no randomized controlled trials directly comparing an 8-week telerehabilitation-supported home exercise program with an unsupervised home exercise programme in adolescents with mild AIS, specifically in terms of posture, pain and health-related quality of life. In this context, the aim of the present study was to examine the effectiveness of a telerehabilitation-supported home-based exercise programme compared with the same home-based exercise programme performed individually without supervision in adolescents with mild AIS. We hypothesized that, although both groups were prescribed the same home-based exercise programme with the same recommended frequency, adolescents with mild AIS receiving an additional once-weekly physiotherapist-supervised telerehabilitation session would demonstrate greater improvements in postural alignment, back pain, body image, and health-related quality of life than those performing the home-based exercises alone. Our study is one of the few that directly examines the effect of supervision on clinical outcomes and has a unique place in terms of comparative evaluation of telerehabilitation with home programs.

## 2. Materials and Methods

### 2.1. Study Design and Participants

This study was conducted to evaluate the effectiveness of telerehabilitation-based exercises performed under the guidance of a physiotherapist in addition to standard home exercise programs used in the treatment of patients with mild AIS. 40 volunteer participants diagnosed with AIS by a physician were included in the study. The required sample size was calculated a priori using GPower software (version 3.1.9.4, Düsseldorf, Germany). Based on posture assessment as the primary outcome and an effect size of 0.88 derived from previous literature, with a type I error of α = 0.05 and 80% statistical power, the minimum sample size was estimated to be 17 participants per group (34 in total) for a two-tailed comparison between groups. To account for possible dropouts, a total of 40 adolescents with AIS were recruited. Participants were evaluated before treatment (baseline) and after the end of the 8-week intervention. All evaluations of AIS patients diagnosed by a physician were performed by research physiotherapists. This study was approved by the Scientific Research Ethics Committee of the Hamidiye University of Health Sciences with the decision numbered 22/268. This study was registered at https://clinicaltrials.gov under the identifier NCT07260383. All participants signed the informed consent form of the Declaration of Helsinki.

Participants were randomized using a computer-generated simple randomization sequence generated by an online randomization tool (https://www.randomizer.org (accessed on 5 December 2025)). After baseline evaluation, an independent administrator opened the next envelope in sequence and assigned participants to their groups. The physiotherapists delivering the telerehabilitation sessions and the researchers conducting outcome assessments were blinded to the allocation sequence.

To minimize selection bias, allocation concealment was ensured through the use of sequentially numbered, sealed, and opaque envelopes, and assignments were made by an independent researcher who was not involved in evaluation or intervention. To reduce measurement bias, all outcome assessments were performed by trained physiotherapists who were blinded to group allocation and who followed a standardized assessment protocol for posture, pain, body image, and quality of life measurements.

### 2.2. Inclusion and Exclusion Criteria

The inclusion criteria for the study were “being between 10 and 18 years old, diagnosed with adolescent idiopathic scoliosis (AIS) and not using a corset, having a Risser stage between 0 and 3 and a Cobb angle between 10° and 25°, individuals with a Type I curve pattern according to the Lenke classification.”

The exclusion criteria were determined as “those with any medical contraindication that may prevent them from doing exercise, patients who have undergone any treatment for scoliosis within the last year, patients who have undergone spinal surgery and patients using a corset, and patients with any mental, neurological and pulmonary disease.”

### 2.3. Clinical Evaluation

The radiological findings, such as Lenke scoliosis classification, Cobb angle, and Risser stage of the participants, were evaluated by the responsible physicians and conveyed to the research physiotherapist. The type was determined according to the Lenke classification based on the structure, location, and size of the curvatures [17]. To determine the degree of deformity of scoliosis in the frontal plane, the Cobb angle was measured on the standing full spine radiograph taken in the anteroposterior direction [18]. The Risser classification, which is graded between 0 and 5, was used to evaluate the ossification of the iliac apophysis [19]. Radiological evaluation was done only at baseline and was not recommended by the physician because of the short following time.

Postural assessment was performed using the New York Posture Assessment Scale [19]. The Visual Analog Scale (VAS) was used to determine the level of pain associated with scoliosis [20]. The body perception of the participants was assessed using the Walter Reed Visual Assessment Scale (WRVAS), which was developed specifically for scoliosis deformity and consists of seven items [21]. In addition, the SRS-22 questionnaire, a 22-item assessment scale developed by the Scoliosis Research Society and specific to scoliosis, was used to measure the quality of life in individuals with scoliosis [22].

### 2.4. Intervention Program

A total of 40 patients included in the study were divided into 2 groups using a simple randomization method. One of these groups was determined as the study group (n = 20) and the other as the control group (n = 20). All patients were evaluated before and after treatment by research physiotherapists in the hospital. Participants included in both groups were given basic information about scoliosis in the hospital, and the importance of the exercises was emphasized. After the first evaluation, home exercises were shown to the control group, and a form containing explanations and pictures of the exercises was shared with the patient in printed form. Participants were asked to apply the exercises at least three times a week and to record the day and duration of each exercise session in an exercise diary. The researcher physiotherapist called these participants every two weeks to follow their compliance with the exercise program. At the end of the eight-week application period, the participants were invited back to the clinic and re-evaluated with the same evaluation methods.

Participants included in the study program group also had the same home exercise program given in the hospital. In addition to these home exercises, the exercises were done online under the guidance of a physiotherapist once a week. An appointment was made for the first telerehabilitation session for the patients in the study group after the evaluation, and the same exercise form was shared with this group. In the online sessions, whether the patient correctly applied the exercise program defined for him/her was monitored and checked online once a week. Supervised telerehabilitation sessions were conducted once per week via the Zoom™ videoconferencing platform (version 5.x; Zoom Video Communications, Inc., San Jose, CA, USA). To ensure standardization, each session followed a structured checklist including posture review, breathing training, exercise execution, and progression criteria. The physiotherapist visually monitored each exercise in real time and provided corrective verbal feedback when compensations or misalignments were observed. Participants were instructed to adjust camera positioning to allow full-body visibility during key exercises. Session duration was approximately 40 min.

The home-based exercise program was structured in two progressive phases over eight weeks. Each session lasted approximately 40 min and was performed at least three times per week. All participants were instructed to complete 8–10 repetitions for 2–3 sets for each exercise, unless otherwise stated.

**First Month Program:**

The first month focused on foundational postural and mobility training. The program included the following:

- Warm-up and cool-down stretching (5–7 min);
- Basic core activation exercises (e.g., pelvic tilts, supine marching, single-leg lifts, bridging);
- Spinal mobility exercises (cat–camel, thoracic rotation, child’s pose variations);
- Side-lying hip abduction and clamshell strengthening;
- Quadruped upper- and lower-limb reaching tasks (bird-dog variations);
- Standing postural alignment and shoulder opening exercises;
- Breathing exercises focusing on diaphragmatic and lateral costal expansion;
- Mirror-based self-correction exercises trained in frontal and sagittal planes.

**Second Month Program:**

In the second month, exercise intensity and neuromotor demands were progressively increased. The program included the following:

- Advanced core stabilization (e.g., bridge with leg extension, dead-bug variations);
- Side-plank progressions;
- Spinal mobility with increased range and segmental control;
- Functional strengthening combining upper and lower extremity movements;
- Thoracic extension and pectoral mobility exercises;
- Improved postural alignment drills performed in standing and mirror feedback tasks;
- Continuation of structured breathing and rib mobility training.

Progression was based on increasing hold duration, number of repetitions, or the complexity of limb movements while maintaining trunk control. The exercise program did not follow any specific method, such as Schroth or SEAS; instead, it reflected the standard physiotherapy approach used clinically for mild AIS in our setting.

### 2.5. Statistical Analyses

Data analyses were conducted using a per-protocol approach. Only participants who completed both baseline and post-intervention assessments were included in the final statistical analysis, as incomplete datasets could not be imputed reliably due to the small sample size. Analyses of the data were performed using the IBM SPSS Statistics for Windows, Version 26.0 (IBM Corp., Armonk, NY, USA) program. Descriptive statistics of the sample for categorical (discrete) data were presented as frequencies and percentages, and for continuous data, the mean, median, standard deviation, min., and max. To determine the differences between the groups before and after the treatment, Mann–Whitney U test and Fisher’s Exact test were used. To analyze the measurement changes in the groups over time, the Wilcoxon Signed Rank test was used for the comparisons within each group. All statistical tests with *p* values less than 0.05 were considered significant. In addition to *p*-values, effect sizes (r) were calculated for all Wilcoxon Signed Rank and Mann–Whitney U tests to improve the interpretability of the findings. Because these non-parametric tests are rank-based, conventional confidence intervals cannot be computed for all outcomes. No adjustments for multiple comparisons were applied, consistent with the exploratory nature of this clinical trial. Effect size values were interpreted according to conventional thresholds, where r = 0.10–0.29 indicates a small effect, r = 0.30–0.49 a moderate effect, and r ≥ 0.50 a large effect [23].

## 3. Results

**Figure 1:**
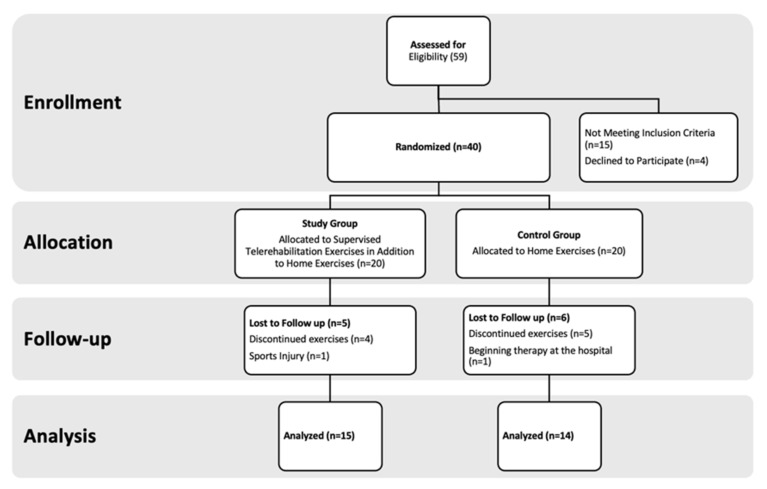
Study Flow Chart.

A total of 40 patients with scoliosis less than 20 degrees and recommended to be followed up with home exercises were included in this study. Participants were randomly divided into two groups as the study group and the control group. Both groups were given a 2-month home exercise program, and unlike the control group, the same exercises were given to the study group online via telerehabilitation under the supervision of a physiotherapist once a week. A total of 15 cases from the study group and 14 cases from the control group completed the study. A total of 11 participants discontinued the study (5 in the study group and 6 in the control group). The analysis was conducted per protocol on the 29 participants who completed both assessments. The flow chart of the study is shown in Figure 1.

Demographic data and scoliosis measurement results of the patients are given in Table 1. There was no difference in demographic data between the groups before the study (*p* > 0.05). There is only a difference between baseline Cobb angle data (*p* < 0.05), and the study group had a greater Cobb angle in the beginning.

The study group followed the online sessions in addition to home exercises. Minor technical issues, such as brief internet interruptions, occurred infrequently but did not prevent the completion of any supervised session. The quality of exercise execution was not numerically quantified; however, the physiotherapist evaluated technique clinically based on alignment, trunk control, and the ability to perform the prescribed repetitions with correct form throughout each session.

**Figure 2:**
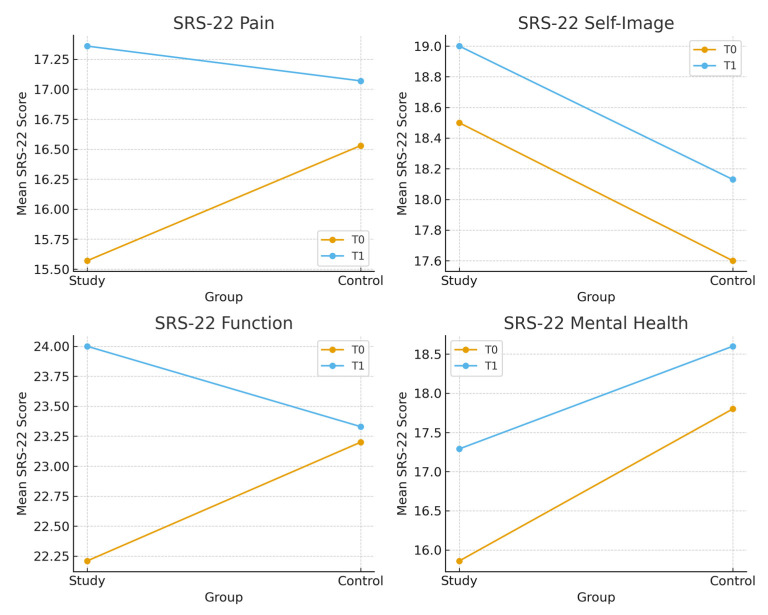
The intergroup differences on SRS-22 subgroups (T0: Before the intervention; T1: After the intervention).

There is a significant difference between the Cobb angles of the randomly determined groups at the beginning (*p* < 0.05). The parameters related to pain, posture, body image and quality of life of both groups were evaluated before and after the intervention. Between-group comparisons revealed no statistically significant differences across these parameters (*p* > 0,05. The intergroup differences on SRS-22 subgroups before and after the intervention are shown in Figure 2.

The study group performed home exercises for an average of 9.14 ± 5.34 sessions, while the control group performed 11.87 ± 7.79 sessions. There was no significant difference between the data obtained from the exercise diaries (*p* < 0.05). There were significant changes in the SRS total score, pain and function sections, and New York posture assessment scale results of the study group (*p* < 0.05). The intragroup differences on outcome measures before and after the intervention are shown in Table 2.

Effect sizes (r) demonstrated that the telerehabilitation group achieved moderate-to-large improvements in key outcomes, including SRS-22 Pain, Function, and Total scores, and NYPAS alignment (r = 0.54–0.76). The home-exercise group showed smaller effect sizes, with only Satisfaction reaching a large effect (r = 0.51). Between-group comparisons demonstrated small-to-moderate effects (r = 0.01–0.45). The updated table now includes effect size values for all non-parametric analyses.

## 4. Discussion

This study was conducted to compare the effects of home-based exercises, with and without telerehabilitation support, on postural alignment (New York Posture Assessment Scale, NYPAS), visually assessed postural deformity (Walter Reed Visual Assessment Scale, WRVAS), back pain and health-related quality of life (SRS-22) in adolescents with AIS. The findings of this preliminary trial suggest that telerehabilitation support may lead to greater improvements in postural alignment, pain level, functional capacity and quality of life than unsupervised home-based exercises; however, these results should be interpreted with caution in view of the small sample size and methodological limitations of the study.

There was no significant difference in terms of demographic and clinical variables at the beginning of the study; only the telerehabilitation group was found to have higher scoliosis degrees at the beginning. This is important in terms of indicating that telerehabilitation can produce effective results even in cases with higher deformities.

Significant improvements were found in the pain, function subscores and total score in the telerehabilitation group in the SRS-22 scale within-group analyses. At the same time, the New York Posture Scale scores also showed significant improvements in this group. In contrast, a significant improvement was observed only in the treatment satisfaction subscore in the home exercise group. Taken together, these preliminary findings suggest that performing the exercises with the supervision of a physiotherapist may not only improve postural alignment but also reduce pain, enhance functional status and positively influence health-related quality of life.

It is seen that supervised exercises are more advantageous in terms of directly applying the technique, strengthening compliance with the exercise and providing motivation in the literature [24]. In a study conducted in 2023, it was reported that there were significant improvements in parameters such as respiratory functions, flexibility and walking distance in individuals with AIS who participated in a telerehabilitation program implemented with a physiotherapist, compared to the group that continued their home exercises individually [15]. In the study of Marin et al. (2021), synchronized telerehabilitation and video-based exercise applications were compared; it was reported that the satisfaction level of the group exercising with a live connection was higher, but continuity was higher in the group working with video [16]. These findings suggest that telerehabilitation models adapted to individual differences may be effective [16]. Similarly, according to the study conducted by Tombak et al. in 2024, individuals with AIS who applied scoliosis-specific exercises 7 days a week for 12 weeks were divided into two separate groups: the supervised group accompanied by a physiotherapist, and the home group that continued their exercises individually [5]. It has been stated that the supervised Schroth exercise program has a more significant effect than the home exercise program in improving the morphometric and cosmetic effects of AIS, but the home exercise program can be applied as an alternative treatment if it is corrected by the therapist at certain intervals [5]. According to the study conducted by Kuru et al. in 2015, it was stated that the group who did Schroth exercises with the supervision of a physiotherapist provided significant improvements in parameters such as lumbar asymmetry, Cobb and rotation angle, and gibbosity height; however, these effects were limited in the group who did individual exercises at home [11]. Similarly, in our study, significant improvements were observed in clinical parameters such as posture, function, and pain in the group who did exercises with telerehabilitation support; these improvements were more limited in the group who did individual exercises at home. These findings support the view that physiotherapist supervision may enhance the effectiveness of exercise programs and represent an important component of the conservative management of scoliosis; however, larger and methodologically stronger trials are needed to confirm this assumption.

The only parameter that showed a significant improvement between pre- and post-treatment in the group that only applied home exercises was the ‘satisfaction with treatment’ sub-dimension in this study. This increase in the satisfaction level with the treatment in the SRS-22 questionnaire suggests that the individual participation of the patients in the exercise process may have had a positive impact on them to some extent. However, the fact that no postural improvement, decrease in pain level or significant change in functional capacities was observed indicates that home exercises carried out only individually have a limited effect on physical outcomes. Therefore, the increase in satisfaction level alone may primarily reflect personal satisfaction associated with engaging in the exercise process, rather than clear physical gains.

In this study, the program duration was set at 8 weeks to allow for measurable changes in postural alignment and related clinical outcomes while maintaining applicability to school-aged adolescents. Studies of posture management and scoliosis-specific exercise for mild to moderate AIS in adolescents also report significant postural and functional improvements with 6–8 weeks of intervention. However, outcomes related to emotional/cosmetic domains, such as body image, appearance satisfaction, and social participation, generally show significant improvement after longer or more intensive interventions [25].

Cosmetic asymmetry caused by scoliosis is an important concern for patients and their families and may lead to poorer psychosocial functioning and body image compared with peers [26,27,28]. Pineda et al. reported that WRVAS scores are significantly associated with the radiological severity of the curve [29]. In our study, which specifically aimed to explore the impact on body image, the lack of significant change in WRVAS scores may suggest that the duration and/or intensity of our exercise program was insufficient to produce measurable improvements in cosmetic perception. Previous work has shown that emotional and cosmetic domains such as self-image, appearance satisfaction and social participation tend to respond more slowly and are more likely to improve after longer-term or more intensive interventions, including prolonged brace treatment or scoliosis-specific exercise programs delivered over 6–12 months, rather than short interventions like ours [30,31]. Moreover, body image in adolescents with scoliosis is influenced not only by the structural deformity itself but also by complex psychosocial factors (e.g., depressive symptoms, peer and family attitudes, social media exposure), which may require multimodal, psychologically informed approaches in addition to physical correction to achieve meaningful changes in WRVAS scores [32].

### Limitations

The limited sample size and the lack of radiological data, such as the Cobb angle, which would cause ethical violations, are among the limitations of our study. The lack of Cobb outcome data prevented direct interpretation of the degree of structural curvature. In addition, our study included a total exercise intervention period of 8 weeks. Short-term interventions may be insufficient to provide significant structural changes, especially in the degree of deformity or aesthetic perception. In future studies, radiological measurements, long-term follow-up, and the use of objective compliance indicators will allow a more comprehensive evaluation of the effectiveness of telerehabilitation.

A further limitation is that exercise execution could not be objectively quantified, particularly in the control group, where real-time supervision was not available. Therefore, inter-individual variability in exercise quality may have influenced functional outcomes.

Another limitation is the absence of pre–post-radiological measurements. Ethical constraints related to repeated exposure to ionizing radiation in adolescents with mild AIS (10–25°), combined with the short 8-week interval between assessments, made additional radiographs inappropriate. According to the clinical protocol in our center, radiographs are not repeated unless progression is suspected. The orthopedic physician responsible for the medical follow-up of the participants did not recommend a radiological reassessment at 8 weeks because no progression was expected within such a short period, and no clinical indications warranted further imaging. Consequently, structural progression could not be evaluated, and the outcomes of the present study reflect functional and postural rather than radiographic changes. Additionally, the groups were not fully homogeneous at baseline in terms of Cobb angle; however, since radiological correction was not a primary outcome and repeated imaging was not ethically indicated, this baseline imbalance is unlikely to have influenced the functional and postural outcomes assessed in this study.

As an additional methodological limitation, postural alignment was assessed only with the observational New York Posture Assessment Scale, which is susceptible to assessor-related bias and does not permit inferences about structural curve correction without radiographic confirmation. In addition, although the SRS-22 is a widely used and validated patient-reported outcome measure in individuals with AIS, the clinical interpretation of changes in its domains in this study was based solely on statistical significance, without a priori consideration of different scale versions or domain-specific thresholds for clinically important change; therefore, these findings should be interpreted with caution.

## 5. Conclusions

This study suggests that home-based exercise programs may provide additional benefits when combined with supervised telerehabilitation in adolescents with mild AIS, particularly in terms of posture, pain, and functional outcomes. However, these findings should be interpreted with caution due to baseline differences between groups, the small sample size, and the methodological limitations inherent to the study design. A more rigorously controlled, larger-scale randomized trial is needed to confirm the effectiveness of supervised telerehabilitation in this population.

## Figures and Tables

**Table 1:** Demographic Data and Scoliosis Measurements.

	Study Group (n = 15)	Control Group (n = 14)	*p*
**Age**	14.71 ± 2.3	14.27 ± 1.67	0.507
**BMI**	20.07 ± 3.48	18.99 ± 2.45	0.394
**Cobb Angle**	18.01 ± 5.34	14.01 ± 4.43	**0.026**
**Sex**	10F/5M	11F/4M	0.617
**Primary Curve**	8L/7T	7S/7L	0.291
**Scoliosis Shape/Type**	6S/9C	8S/6C	0.566

Bold: it is for the significant results (*p* < 0.05).

**Table 2:** Intragroup differences on outcome measures before and after the intervention.

	Study Group T0 (Mean ± SD)	Study Group T1 (Mean ± SD)	*p*	Effect Size (r)	Control Group T0 (Mean ± SD)	Control Group T1 (Mean ± SD)	*p*	Effect Size (r)
**Pain Severity (VAS)**	1.57 ± 0.65	1.64 ± 0.63	0.705	−0.10	3.27 ± 2.22	2.60 ± 2.29	0.234	−0.31
**Pain Frequency**	2.93 ± 2.24	2.36 ± 2.68	0.395	−0.23	3.00 ± 2.45	2.07 ± 1.98	0.159	−0.36
**Walter Reed Visual Assessment Scale**	11.50 ± 3.55	12.21 ± 2.67	0.397	−0.23	11.67 ± 3.64	11.07 ± 2.79	0.384	−0.22
**Newyork Posture Scale**	52.86 ± 15.16	61.43 ± 15.37	**0.004**	**−0.76**	49.33 ± 14.38	53.00 ± 18.01	0.590	−0.14
**SRS-22 Pain**	15.57 ± 3.59	17.36 ± 1.95	**0.044**	**−0.54**	16.53 ± 2.26	17.07 ± 2.09	0.074	−0.27
**SRS-22 Self Image**	18.50 ± 4.49	19.00 ± 3.66	0.805	−0.07	17.60 ± 2.87	18.13 ± 1.69	0.505	−0.17
**SRS-22 Function**	22.21 ± 2.12	24.00 ± 1.24	**0.007**	**−0.72**	23.20 ± 1.42	23.33 ± 1.76	0.791	−0.07
**SRS-22 Mental Health**	15.86 ± 4.85	17.29 ± 4.98	0.157	−0.38	17.80 ± 4.09	18.60 ± 3.64	0.318	−0.26
**SRS-22 Satisfaction/Dissatisfaction**	8.36 ± 1.74	9.07 ± 1.07	0.070	−0.48	7.93 ± 2.09	8.87 ± 1.19	**0.046**	**−0.51**
**SRS-22 Total**	74.8 ± 7.43	77.86 ± 9.65	**0.023**	**−0.61**	77.6 ± 7.72	77.60 ± 7.73	0.074	−0.46

## Data Availability

The data that support the findings of this study are available from the corresponding author upon reasonable request.

## Abbreviations

The following abbreviations are used in this manuscript:

AIS	Adolescent idiopathic scoliosis
VAS	The Visual Analog Scale
WRVAS	Walter Reed Visual Assessment Scale
SRS-22	Scoliosis Research Society-22
